# Recurrent intracerebral hemorrhage as a rare presentation of mycotic aneurysm secondary to infective endocarditis: A case report

**DOI:** 10.1016/j.ijscr.2025.111591

**Published:** 2025-06-30

**Authors:** Ram Prasad Subedi, Nikunja Yogi, Suraj Thulung, Suresh Bishokarma, Ajit Thakali, Sadikshya Yogi

**Affiliations:** Department of Neurosurgery, Upendra Devkota Memorial National Institute of Neurological and Allied Sciences (UDM-NINAS), Bansbari, Kathmandu, Nepal

**Keywords:** Mycotic aneurysm, Recurrent intra-cerebral hemorrhage, Infective endocarditis, Case report

## Abstract

**Introduction and importance:**

Mycotic aneurysms are a rare but serious complication of systemic infections such as infective endocarditis. When ruptured, they carry a high risk of mortality and are an uncommon cause of intracerebral hemorrhage.

**Case summary:**

A 54-year-old male, initially presented with a right Middle Cerebral Artery (MCA) territory infarct and was managed conservatively. He later developed an intracerebral hemorrhage requiring surgical evacuation, followed by a second hemorrhagic event. Digital Angiography revealed a ruptured mycotic pseudoaneurysm in the right MCA M4 segment; he was managed conservatively with antibiotics and recovered well by three- month follow up.

**Discussion:**

Mycotic aneurysms are though rare but can be managed medically with early detection and prompt diagnosis. Multidisciplinary team of Neurologists, neurointerventionist, cardiologists, radiologists, clinical psychologist discussion is needed for timely diagnosis of such illness and appropriate treatment. Patient with hemodynamically stable status, small hematoma, can be managed conservatively with antibiotics whereas massive hematoma, ruptured aneurysm needs surgical or endovascular treatments.

**Conclusion:**

Prompt and thorough radiological assessment-including non-contrast CT of the brain and cerebral angiography, coupled with detailed cardiac evaluation such as echocardiography, is pivotal in the early detection of mycotic aneurysm in patients presenting with intracerebral hemorrhage. Such a comprehensive diagnostic approach not only facilitates timely intervention but also significantly contributes to improved clinical outcomes. Treatment should be tailored according to the clinical condition of each patient.

## Highlights

• Mycotic aneurysms are very rare but potentially fatal complications of infective endocarditis.
• Hemodynamically and neurologically stable patient can be managed conservatively with antibiotics.
• Massive hemorrhage and clinical deterioration warrant surgical/endovascular procedure.

## 1 Introduction

Mycotic aneurysms (MAs), also referred to as infective or microbial aneurysms, are uncommon inflammatory lesions affecting the neurovascular system [1]. Mycotic aneurysms constitute a rare subset of intracranial aneurysms, comprising roughly 0.7 % to 6.5 % of all similar cases [1,2]. MAs have a distinctive natural history, and a tendency to form at terminal artery branches. They are linked to substantial morbidity and death, which can range from 60 % to 90 % in previous case studies and 12 to 32 % in more current research, since their spontaneous rupture causes subarachnoid and intracerebral hemorrhage [[3], [4], [5]]. More than 80 % of mycotic aneurysms in the brain are specifically caused by cardiac bacterial endocarditis/infective endocarditis. Approximately 2 % to 4 % of patients with Infective endocarditis develop mycotic aneurysm, and up to 30 % of them experience neurological signs and symptoms [1,2,4,6]. Infective causes include mainly septic emboli from left-sided cardiac bacterial endocarditis (*Staphylococcus aureus* and viridians group streptococci) along with extravascular sources of infection like meningitis, post neurosurgical infections, cerebritis, and paranasal sinuses infection [7]. They pose a significant challenge to surgical treatment due to systemic comorbidity and their occurrence in small vessels. Endovascular treatment is associated with good outcomes. In this case report, we highlight the clinical dilemma associated with the diagnosis and investigation of mycotic aneurysms, as well as the management and patient outcomes. This case report has been reported in the line with the SCARE Criteria [8].

## 2 Case history

A 54-year-old right-handed, married male with a history of intravenous drug use presented to the emergency department of a local center with complaints of left-sided body weakness and deviation of the mouth to the right, both lasting for one day. These symptoms were accompanied by slurred speech. On neurological examination, his Glasgow Coma Scale (GCS) score was 12/15, with spontaneous eye opening, confused verbal responses, and withdrawal to pain. Muscle tone was increased, and motor power was reduced to 3/5 in the left upper and lower limbs, while the right-sided power was preserved at 5/5. Plantar reflexes were flexor bilaterally, and deep tendon reflexes were hyperactive (Grade III) on the left side. Sensory function was intact.

**Fig. 1:**
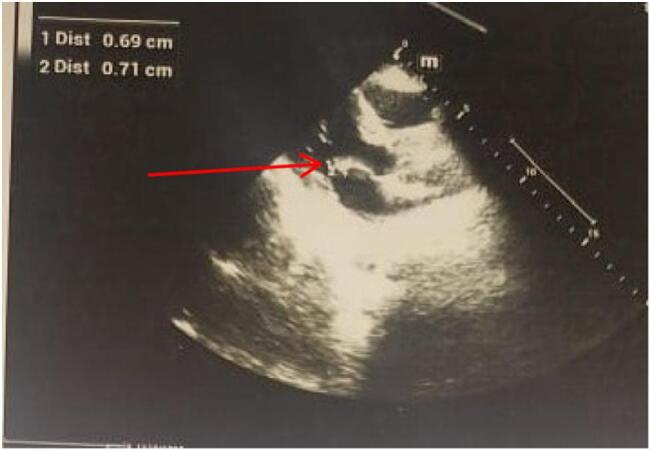
Echocardiography showed thickened anterior mitral valve leaflet with no evidence of vegetation (red arrow).

A non-contrast head CT scan revealed a subacute infarct involving the right frontal, temporal, and parietal lobes, corresponding to the right middle cerebral artery (MCA) territory. Transthoracic echocardiography (Fig. 1) showed a thickened anterior mitral valve leaflet with mild to moderate mitral regurgitation but no visible vegetations. Carotid and vertebral artery Doppler studies were normal. Incidentally, the patient tested positive for hepatitis C antibodies. He was managed medically with oral aspirin (150 mg once daily) and atorvastatin (20 mg once daily). After five days of hospitalization, he was discharged with continuation of the prescribed medications.

Two weeks later, the patient developed a sudden-onset headache and left-sided weakness, both lasting for six hours, along with two episodes of vomiting. There was no reported history of loss of consciousness, seizures, trauma, or bladder/bowel incontinence. Upon examination, he was alert and oriented (GCS 15/15), with increased muscle tone and reduced power (3/5) in the left limbs. Right-sided motor function remained intact. Pupils were bilaterally reactive, sensory function was preserved, and the left plantar reflex was extensor with hyperreflexia (Grade III deep tendon reflexes) on the same side.

**Fig. 2:**
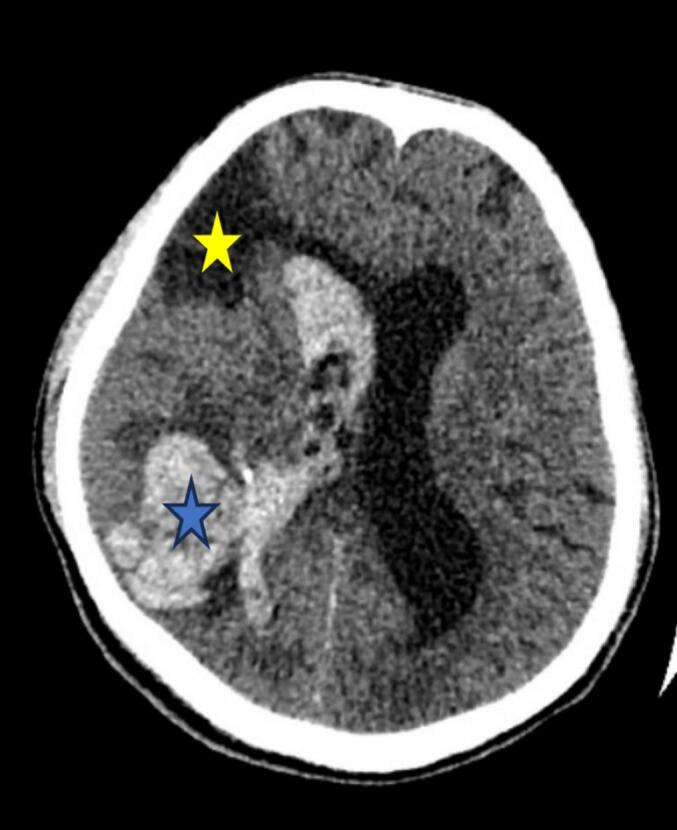
A non-contrast head CT scan showing right parietal ICH (blue star) with intra-ventricular extension (yellow star).

He was admitted to the internal medicine department and started on levetiracetam (500 mg once daily), citicoline (500 mg three times daily), and antiviral therapy (Sofosbuvir/Velpatasvir 400/100 mg once daily). A non-contrast head CT scan (Fig. 2), performed due to a transient decrease in GCS, revealed a right parietal intracerebral hemorrhage with intraventricular extension. On the same day, he underwent an emergency right parietal craniotomy with hematoma evacuation and lax duroplasty under general anaesthesia. The surgery lasted five hours. Intraoperative findings included a swollen but pulsatile brain, and approximately 30 ml of hematoma was evacuated. The postoperative course was uneventful, and he was discharged 11 days later with a GCS of 15/15 and stable vital signs. The surgical site was healthy.

One month later, he presented to our emergency department with a two-day history of dull, aching frontal headaches, sudden in onset, associated with two non-projectile episodes of vomiting. His relatives reported a transient period of decreased consciousness lasting 3–4 h. At presentation, the patient was afebrile and denied trauma, limb weakness, or bladder/bowel incontinence. Neurological examination revealed a GCS of 15/15, preserved cognition and motor power (5/5 in all limbs), intact sensation, bilaterally reactive pupils (2 mm), flexor plantar reflexes, and normal (Grade II) deep tendon reflexes.

**Fig. 3:**
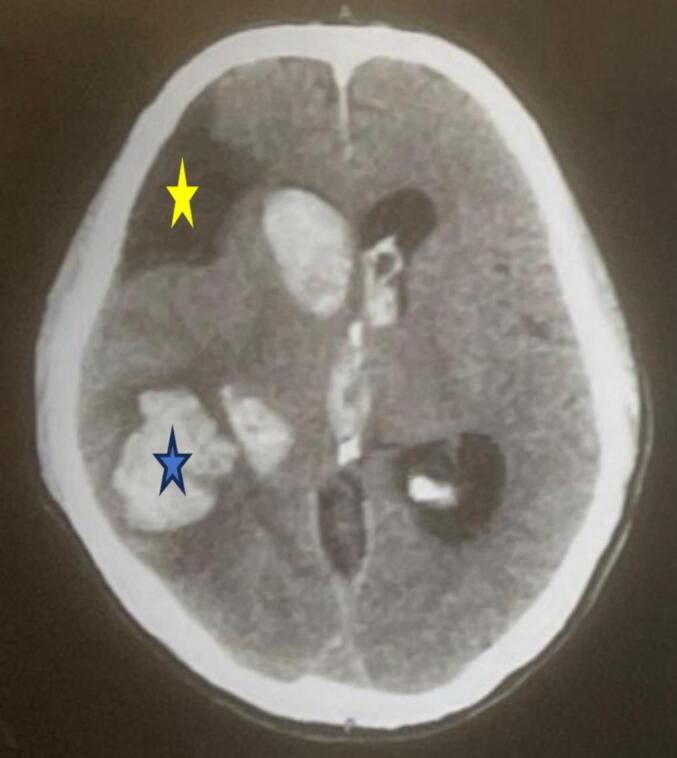
A non-contrast head CT scan shows right parietal intracerebral hematoma with intraventricular extension (blue star) with old infarct over right frontal region (yellow star).

A repeat non-contrast head CT scan (Fig. 3) demonstrated an intraparenchymal hemorrhage in the right parietal lobe with intraventricular extension. In addition, chronic infarct areas with encephalomalacia and gliosis were noted in the watershed territories of the right anterior cerebral artery (ACA) and MCA. Blood cultures were negative for microbial growth.

A multidisciplinary team—comprising neurosurgeons, neurologists, neurointerventionist, cardiologists, neuroradiologists, and clinical psychologists—was engaged for comprehensive case evaluation and management. The patient was admitted under neurosurgical care for hemorrhagic stroke, with neurologists addressing his previous ischaemic stroke. The neurointerventionist performed digital subtraction angiography (DSA), while the cardiologist conducted echocardiographic evaluation, which indicated infective endocarditis. The neuroradiologist reviewed all prior imaging, and the clinical psychologist provided psychological support.

**Fig. 4:**
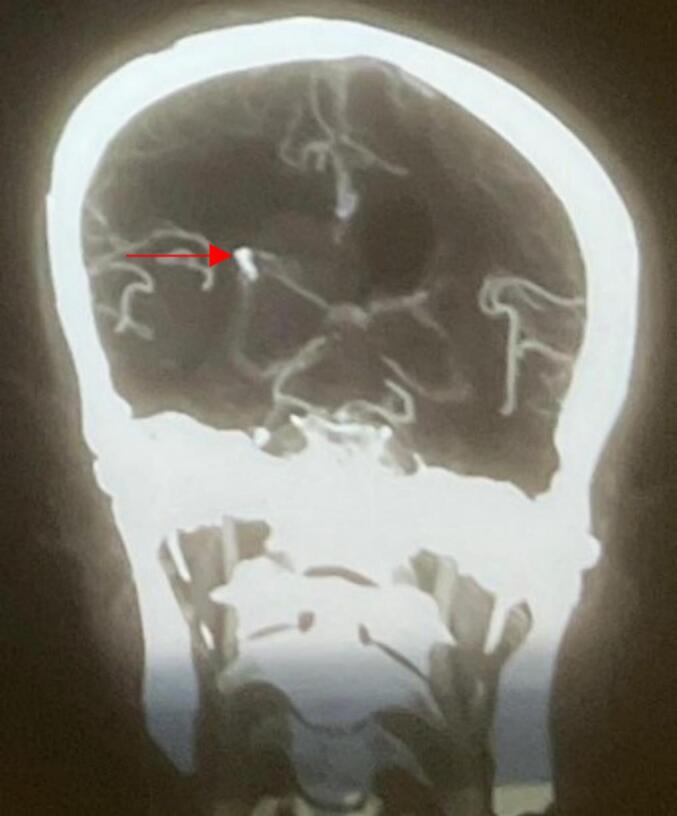
CT cerebral angiography shows distal right middle cerebral artery suspected aneurysm (red arrow).

**Fig. 5:**
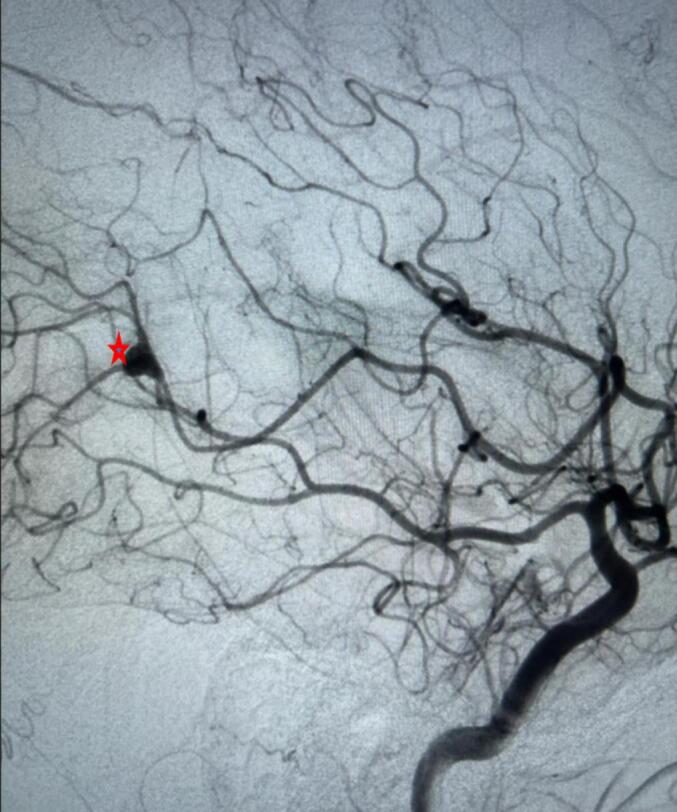
Digital subtraction angiography of cerebral vessels shows distal right middle cerebral artery mycotic aneurysm (red star).

**Fig. 6:**
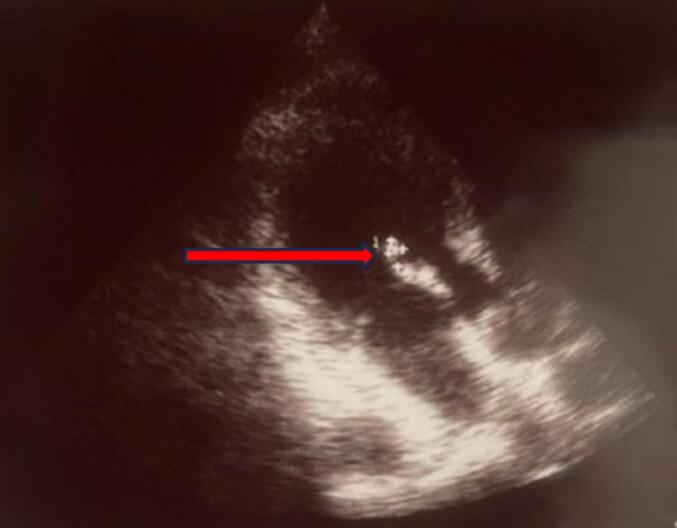
Echocardiography shows thickened anterior mitral valve leaflet with a vegetation of 7 × 4 mm with moderate eccentric mitral regurgitation (red arrow).

CT cerebral angiography (Fig. 4) revealed an intraparenchymal hemorrhage in the right temporoparietal region with intraventricular extension and a 4 mm midline shift. DSA of the right internal carotid artery identified a pseudoaneurysm measuring approximately 4.5 × 4.1 mm arising from the parietotemporal branch of the M4 segment of the right MCA (Fig. 5). Echocardiography (Fig. 6) showed a thickened anterior mitral valve leaflet with a suspected 7 × 4 mm vegetation, moderate eccentric mitral regurgitation, and a preserved left ventricular ejection fraction of 60 %.

The patient was admitted to the High Dependency Unit and managed conservatively with intravenous levetiracetam (1 g twice daily), piperacillin/tazobactam (4.5 g four times daily), ceftriaxone (2 g once daily), gentamicin (150 mg once daily), and continuation of antiviral therapy. He remained clinically stable throughout the 14-day hospital stay and patient was discharged on day 15 in good condition, with stable vital signs and no new neurological deficits.

At a three-month follow-up, the patient demonstrated significant clinical improvement. His GCS remained 15/15, with a modified Rankin Scale (mRS) score of 1, indicating minimal neurological disability. Follow-up investigations—including complete blood count, C-reactive protein, and repeat blood cultures—were all within normal limits.

## 3 Discussion

Infective endocarditis (IE) remains a challenging condition to manage, with a one-year mortality rate approaching 30 % [1,3,9,10]. Neurological complications, particularly cerebral embolism, occur in 20–40 % of cases and significantly contribute to poor outcomes [2,11]. Among these, mycotic aneurysms (MAs)—though relatively rare with an incidence of 2–3 %—pose a significant risk due to their high rupture rates and associated mortality of up to 80 % if ruptured and 30 % if left untreated. Most MAs are located in the anterior circulation and often present with nonspecific symptoms such as headache, fever, vomiting, altered mental status, and focal neurological deficits, or may manifest dramatically as intracerebral hemorrhage [5].

The “vasa vasorum theory” explains the pathogenesis of MAs as microbial infiltration leading to arterial wall destruction [1,3,9,11]. In our case, the patient presented with a sudden-onset headache and vomiting, with neuroimaging confirming an intracerebral hemorrhage and intraventricular extension. This aligns with existing literature emphasizing the utility of CT angiography and MRI for early detection. While non-invasive imaging plays a crucial diagnostic role, digital subtraction angiography remains the gold standard due to its superior anatomical resolution and ability to monitor aneurysm evolution [3].

Historical case reports support a range of management strategies. Kuo et al. described a patient with mitral valve endocarditis who later developed a ruptured cerebral aneurysm requiring surgical clipping [1]. In contrast, our patient, who remained hemodynamically stable and showed no signs of raised intracranial pressure, responded well to conservative treatment with antibiotics alone. Similarly, Musthafa et al. reported a young female patient with IE and a mycotic aneurysm who improved without surgical intervention [11]. Our case mirrors this conservative approach, with continued improvement and no recurrence at the 3-month follow-up.

A comprehensive cardiac workup—including transthoracic or transesophageal echocardiography—is essential to identify potential cardiac sources of emboli or valvular lesions. This integrative neurocardiac assessment is central to managing patients with suspected IE and secondary neurological involvement. Early identification of MAs allows for timely antimicrobial therapy and surgical or endovascular planning when indicated [7,9].

Singla et al. reported another similar case involving a mitral valve abscess and cerebral aneurysm, managed with heart failure medications and intravenous antibiotics, further supporting the variability in presentation and management approaches [5].

In this case, the decision to pursue conservative management following the diagnosis of a ruptured pseudoaneurysm was guided by several clinical and radiological considerations. The pseudoaneurysm, measuring approximately 4.5 × 4.1 mm, was in the M4 segment of the right middle cerebral artery—an anatomically distal and surgically challenging location. According to existing literature, distal MCA aneurysms, particularly when small (<5 mm) and not associated with mass effect or worsening neurological status, may be managed medically in carefully selected, hemodynamically stable patients [[1], [2], [3]]. At the time of the second hemorrhagic event, the patient maintained a Glasgow Coma Scale score of 15/15, had no signs of raised intracranial pressure or clinical deterioration, and showed no new focal deficits. These factors, combined with the lack of aneurysm enlargement or active contrast extravasation on angiography, supported the feasibility of conservative management with intravenous antibiotics targeting the underlying infective endocarditis [10].

The initial decision to perform surgical evacuation during the first hemorrhagic episode was prompted by acute neurological deterioration, specifically a significant drop in GCS and clinical signs of raised intracranial pressure. Imaging at that point demonstrated a sizable hematoma with intraventricular extension, mandating urgent decompression. In contrast, the second episode occurred with preserved consciousness, smaller hematoma volume, and no radiological indicators of impending herniation, thus justifying a shift toward non-operative management. This clinical course reflects the importance of individualized, condition-based decision-making in patients with mycotic aneurysms and aligns with published recommendations favouring conservative treatment in stable cases [1,4,5].

This case underscores the importance of maintaining a high index of suspicion for mycotic aneurysms in patients presenting with hemorrhagic stroke and underlying infective endocarditis. Early neuroimaging—particularly CT angiography and/or digital subtraction angiography plays a crucial role in diagnosis. Multidisciplinary collaboration involving neurosurgery, cardiology, infectious disease, and radiology is essential to guide individualized treatment decisions. Conservative management with targeted antibiotics can be effective in hemodynamically stable patients without signs of increased intracranial pressure. However, vigilant follow-up is critical to detect recurrence or progression. Future prospective studies with larger cohorts are needed to establish standardized guidelines for the diagnosis and management of this rare but serious complication.

## 4 Conclusion

Mycotic aneurysms are infrequent but fatal occurrence and should maintain a high clinic attention as it tends to present as intracerebral hemorrhage. Early neuroimaging with non-contrast CT scan of head and cerebral angiography is crucial for early identification, particularly when clinical features are atypical. Multidisciplinary care is necessary for comprehensive evaluation and management. Echocardiography plays an important role in the detection of possible source of cardiac origin. Personalized therapeutic strategies for patients with personalized clinical status and strict follow-up are key to outcomes improvement and complications reduction.

### 4.1 Strength of the study

The strength of the study lies in the multidisciplinary team approach employed in managing the patient. This case represents a rare and clinically significant instance of recurrent intracerebral hemorrhage secondary to a mycotic aneurysm in the context of infective endocarditis.

### 4.2 Limitation

The Limitation of this case report is that the patient follow-up was based solely on clinical examination, as radiological investigations were not feasible during the follow-up period.

## CRediT authorship contribution statement

Ram Prasad Subedi: Conceptualization, writing original draft, manuscript review
Nikunja Yogi: Manuscript review, resources
Suraj Thulung: Supervision, manuscript review
Suresh Bishokarma: Supervision, literature review, resources
Ajit Thakali: Manuscript review, supervision
Sadikshya Yogi: Writing original draft, resources.

## Consent

Written informed consent was taken from the patient for relevant images, and for publication. a copy of the written consent is available for review by the Editor-in-Chief of this journal on request.

## Ethical approval

It is exempted in our Institution and ethical approval was taken from the intuitional review committee.

## Guarantor

Ram Prasad Subedi.

## Research registration number

None.

## Funding

This study is not funded by any organization or institution.

## Declaration of competing interest

There is no conflict of interest.

## References

[1] Kuo I., Long T., Nguyen N., Chaudry B., Karp M., Sanossian N. (2010). Ruptured intracranial mycotic aneurysm in infective endocarditis: a natural history. Case Rep. Med..

[2] Charan B.D., Gaikwad S.B., Agarwal S., Jain S. (Sep 30 2024). Endovascular treatment of mycotic intracranial aneurysms: a series of three cases with institutional treatment algorithm. Asian J Neurosurg..

[3] Morris N.A., Matiello M., Lyons J.L., Samuels M.A. (Oct 2014). Neurologic complications in infective endocarditis: identification, management, and impact on cardiac surgery. Neurohospitalist.

[4] Peters P.J., Harrison T., Lennox J.L. (2006). A dangerous dilemma: management of infectious intracranial aneurysms complicating endocarditis. Lancet Infect. Dis..

[5] Singla V., Sharma R., Nagamani A.C., Manjunath C.N. (Jun 28 2013). Mycotic aneurysm: a rare and dreaded complication of infective endocarditis. Case Rep. Dermatol..

[6] Chun Jay Y., Smith Wade, Halbach Van V., Higashida Randall T., Wilson Charles B., Lawton Michael T. (June 2001). Current multimodality management of infectious intracranial aneurysms. Neurosurgery.

[7] Kannoth S., Iyer R., Thomas S.V., Furtado S.V., Rajesh B.J., Kesavadas C., Radhakrishnan V.V., Sarma P.S. (May 15 2007). Intracranial infectious aneurysm: presentation, management and outcome. J. Neurol. Sci..

[8] Kerwan A., Al-Jabir A., Mathew G., Sohrabi C., Rashid R., Franchi T., Nicola M., Agha M., Agha R.A. (2025). Revised Surgical CAse REport (SCARE) guideline: an update for the age of artificial intelligence. Premier Journal of Science.

[9] Phuong L.K., Link M., Wijdicks E. (Nov 2002). Management of intracranial infectious aneurysms: a series of 16 cases. Neurosurgery.

[10] Habib G., Hoen B., Tornos P. (2009). ESC Committee for Practice Guidelines endorsed by the European Society of Clinical Microbiology and Infectious Diseases (ESCMID) and the International Society of Chemotherapy (ISC) for Infection and Cancer. Guidelines on the prevention, diagnosis, and treatment of infective endocarditis (new version 2009): the Task Force on the Prevention, Diagnosis, and Treatment of Infective Endocarditis of the European Society of Cardiology (ESC). Eur. Heart J..

[11] Musthafa I., Kandel D., Rajlawot K., Neupane N.P., Sitaula A. (Aug 1 2022). Infective endocarditis complicated by cerebral abscess and mycotic intracranial aneurysm: a case report. Radiol Case Rep..

